# Comparison of the shaping ability of RaCe, FlexMaster, and ProFile nickel-titanium instruments in severely curved root canals

**DOI:** 10.4317/jced.52838

**Published:** 2016-12-01

**Authors:** Lea Burkhardt, Frank Weidmann, Stefan Rüttermann, Susanne Gerhardt-Szep

**Affiliations:** 1 Dr. med. dent, Master of Science Endodontics (M.Sc.), Department of Operative Dentistry, Center for Dentistry and Oral Medicine, Medical Faculty, Goethe University, Frankfurt am Main, Germany; 2 Dr. med. dent, Private Practice, Kronberg, Germany; 3 Full Professor in Dentistry, Head of Department of Operative Dentistry, Center for Dentistry and Oral Medicine, Medical Faculty, Goethe University, Frankfurt am Main, Germany; 4 PD Dr. med. dent and Master of Medical Education (MME), Department of Operative Dentistry, Center for Dentistry and Oral Medicine, Medical Faculty, Goethe University, Frankfurt am Main, Germany

## Abstract

**Background:**

This *in vitro* study compared the shaping ability of RaCe, FlexMaster, and ProFile rotary nickel-titanium instruments in severely curved root canals of extracted teeth.

**Material and Methods:**

Sixty maxillary molars with curvatures ranging from 25° to 65° were embedded in a muffle system and portioned into five horizontal sections (thickness 1.2 mm), starting from the apex. Canals were divided into three groups (n = 20, each) and were prepared with RaCe, FlexMaster, or ProFile rotary nickel-titanium instruments and the TriAuto ZX handpiece using a crown-down preparation technique. We evaluated the difference between pre- and postoperative root canal cross-sections, loss of working length, instrument failure, and preparation time. The root canal area before and after the intervention was determined using an area-measuring software. The data were analyzed statistically using a one-way ANOVA followed by a Kruskal-Wallis multiple-comparison Z-value test.

**Results:**

Specimens treated with FlexMaster showed the greatest change from preoperative cross-sections, followed by RaCe and ProFile. The cross-sectional changes induced by RaCe and FlexMaster preparation differed significantly from those produced by ProFile. Loss of working length, instrument failure, and preparation time did not differ significantly between the groups.

**Conclusions:**

Root canal preparation with the three instruments did not lead to any significant alteration of the original root anatomy or working length. Thus, we conclude that RaCe, FlexMaster, and ProFile instruments are of comparable efficiency and usefulness in the preparation of severely curved root canals.

** Key words:**Endodontics, root canal preparation, rotary, extracted teeth, nickel-titanium.

## Introduction

The preparation of severely curved root canals remains challenging, even for experienced endodontists. Since the 1990s, new rotary nickel-titanium (Ni Ti) instruments have continuously been developed for the safe as well as easier and faster use compared to the manual stainless steel instruments in the preparation of even severely curved root anatomy (1,2). Successful endodontic treatment hinges mainly on preserving the original anatomy of the root canal whilst avoiding instrument fractures and iatrogenic preparation errors, such as loss of working length, zipping, or ledging (3,4). To avoid such preparation errors and apical extrusion of infected debris, the crown-down technique is frequently one possible method in the treatment of curved root canals (5). This technique forms the conceptual basis of the RaCe (FKG Dentaire, La-Chaux-de-Fonds, Switzerland), FlexMaster (VDW, Munich, Germany), and ProFile (Maillefer, Ballaigues, Switzerland) Ni-Ti files whose instrumental designs differ as detailed in  Table 1. The RaCe instrument system differs from the ProFile and FlexMaster instruments by the presence of alternating cutting edges that named this system (i.e., reamer with alternating cutting edges). According to the manufacturer’s information, blockage of the root canal is prevented by alternating curved and straight cutting parts of the instrument used. In contrast, the passive ProFile instruments possess a flat area, the so-called radial lands, behind their blades thus providing a large contact area between the blades and root canal wall leading to increased friction and torque values that may lead to fractures (6). The cross-section of the FlexMaster instrument is convex, and the three active blades have a negative cutting angle similar to that of the K-file. Many studies document good results for the FlexMaster in terms of preserving the original anatomy of the root canal (7-9). Previous studies have assessed the shaping ability of the three instrument types in the preparation of simulated curved root canals (10,11). Schäfer and Oitzinger reported significantly better cutting efficiency for RaCe and FlexMaster instruments than for the ProFile instrument (10), whereas Schirrmeister *et al.* found more effective cleaning of root canal walls and reduced loss of working length with the RaCe instrument in comparison with the FlexMaster und ProFile instruments in simulated curved root canals (11). Two other studies comparing the three instrument types in extracted teeth focused on the presence of smear layer (12) and apically extruded debris after the preparation (13). To our knowledge, no studies comparing the shaping ability of the RaCe, ProFile, and FlexMaster systems in natural teeth have been published so far. Therefore, the present *in vitro* study in extracted human upper molars aimed to compare the performance of the RaCe, FlexMaster, and ProFile instruments in the preparation of severely curved root canals under conditions as close as possible to those encountered in the clinical situation. Our null hypothesis assumed that the three differently designed instrument types are not associated with any differences in the postoperative cross-section of root canals, loss of working length, frequency of fracture, or preparation time.

**Table 1:**
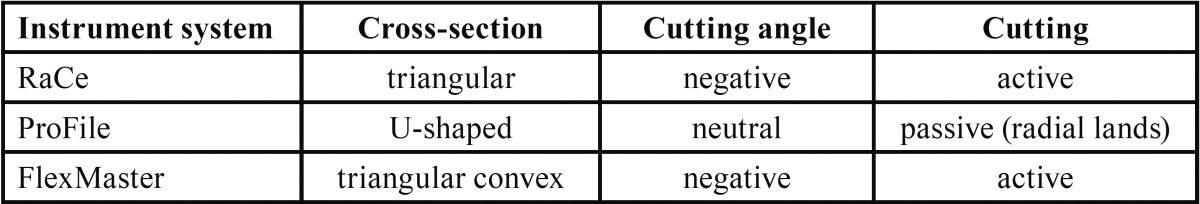
Design characteristics of the three instruments used.

## Material and Methods

For this study, we selected 60 extracted upper molars whose mesial root canals had a curvature of at least 25°. The angle of the curvature was determined using the method of Schneider (14). For sample preparation, we used a modified Bramante technique (15). The molars were trepanned with a diamond bur of ISO size 14 (Gebr. Brassler, Lemgo, Germany). Each tooth was fixed separately on a 2 x 2 x 4 cm aluminium cuvette using a metal wire to avoid sinking of the tooth during embedding in a fast-hardening, cold-polymerizing resin (Technovit 4004, Heraeus Kulzer GmbH, Wertheim, Germany). After hardening, the resin block was removed from the cuvette and marked with a diagonal groove on one side to facilitate the subsequent determination of the cutting plane. To achieve a uniform surface, a 9 mm layer of the embedded tooth was trimmed off coronally to the apex. Starting from the apex, the tooth-resin blocks were sectioned into five horizontal slices (thickness 1.5 mm each), using a saw microtome (SP 1600, Leica, Wetzlar, Germany). The final slice thickness amounted to 1.2 mm, owing to the saw-blade thickness of 280 µm and the modest vertical unevenness of approx. ± 50 µm.

Before and after preparing the root canals, the mesial parts of the root cross-sections were digitized from their top and bottom sides at a magnification of 1.25 x 6.3 x 2.0, using a CCD camera (CF11/2, Kappa, Gleichen, Germany) on a macroscope (M410, Wild, Heerbrugg, Switzerland). An angular specimen holder attached to the object stage ensured that the position of taking images of the section planes under the macroscope was reproducible.

**Figure 1:**
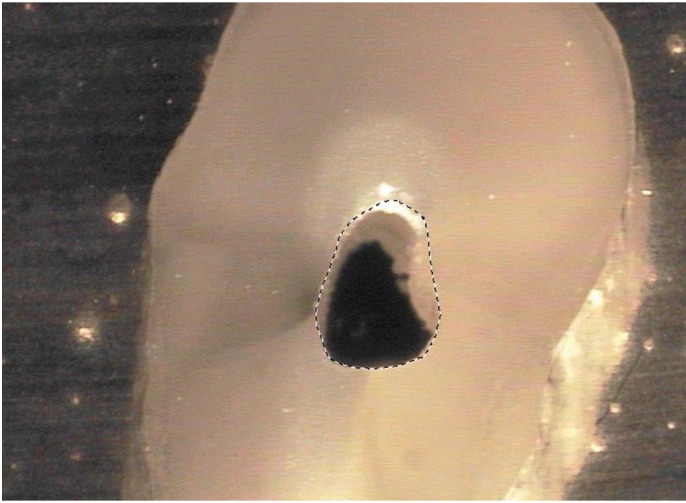
Evaluation-Screenshot of the digitized root canal cross-sectional area with “Image 2000”, showing step 2 “Select a region of interest /ROI with the polygon tool” (Workflow: Step 1 Viewing an image >Step 2 Defining image properties / Setting the scale > Step 3 Image analysis / Measuring ROI).

To calculate the change of canal area after the preparation of the specimens (i.e., canal area difference = canal area after minus canal area before), the digitized cross-sectional areas of the root canals were quantified using the software Image 2000 (SDS NASA Goddard Space Flight Center Code 588, Greenbelt, USA) according to users guide Version 1.1 (Fig. 1).

Endodontic working length was defined as the length from the plane coronal surface to the radiographic apex minus 1 mm. To quantify the working length, we inserted instruments of known length (ISO size 15) into the root canals of the tooth-resin specimens and obtained single-tooth images. Instrument length was measured and corrected using the software Merlin 2.1 (mdc-medical digital concepts GmbH & Co. KG, Neu-Ulm, Germany), based on the radiologically visible distance between the instrument tip and radiographic apex minus 1 mm. Loss of working length after the preparation of root canals was determined using the same procedure, but this time we used the apical master file ISO size 40/02 of the particular instrument system being tested.

The tooth-resin specimens (n = 60) were randomly allocated to one of three groups, each consisting of 20 root canals. The mean angles of curvature amounted to 41° for the RaCe group, 41° for the FlexMaster group, and 40° for the ProFile group. After canal enlargement with a Peeso drill of ISO size 160 (VDW, Munich, Germany), the canals in the three groups were prepared with the crown-down technique using either RaCe, FlexMaster, or ProFile Ni-Ti instruments in combination with the endodontic handpiece TriAuto ZX (Morita, Dietzenbach, Deutschland) at 300 U/min in manual mode. The manufacturers of the various instruments recommend the use of variable file sequences, depending on the clinical situation. We standardized instrument sequencing for the three instrument systems as follows: 06/30, 04/30, 04/25, 02/20, 02/25, 02/30, 02/35, and 02/40. Before starting the procedure and after every change of instruments, the specimens were flushed with 1 mL of 2.5% sodium hypochlorite solution.

Statistical analysis of the study variables, i.e., postoperative cross-section of root canals, loss of working length, frequency of instrument fracture, and preparation time, was done by a one-way analysis of variance (ANOVA; NCSS 97). Significant differences were determined using the nonparametric Kruskal-Wallis multiple-comparison Z-value test according to Bonferroni. In addition, means ± standard deviations (SD), were evaluated.

## Results

**Figure 2:**
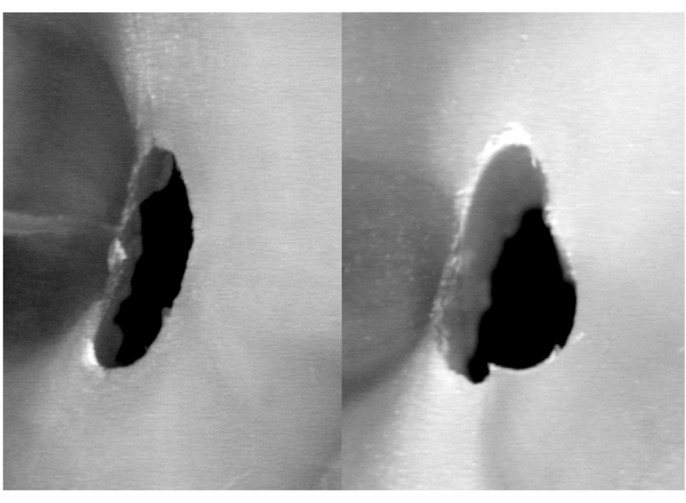
Specimen of the FlexMaster group before (left) and after (right) preparation.

**Figure 3:**
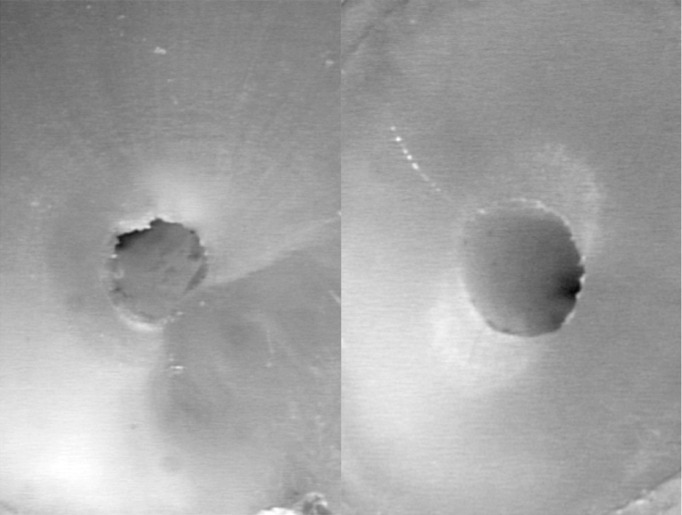
Specimen of the RaCe group before (left) and after (right) preparation.

**Figure 4:**
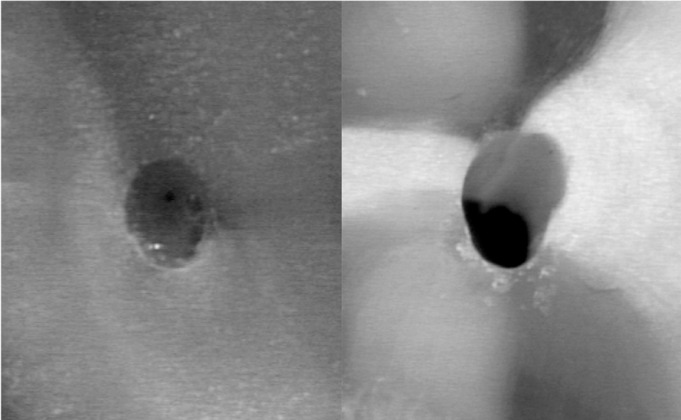
Specimen of the ProFile group before (left) and after (right) preparation.

When comparing the postoperative cross-sections of root canals for all section planes measured, the FlexMaster group showed the most extensive loss of material (0.19 ± 0.10; mean ± SD), followed by the RaCe (0.16 ± 0.10) and ProFile (0.14 ± 0.15) groups (Figs. 2-4). These differences were statistically significant for RaCe and FlexMaster compared to ProFile. Similarly, the largest difference in areas of individual canal sections (coronal, at the start of the curvature, and in the apical canal sections) was seen with the FlexMaster, while use of the ProFile instruments resulted in the smallest material loss ( Table 2).

**Table 2:**
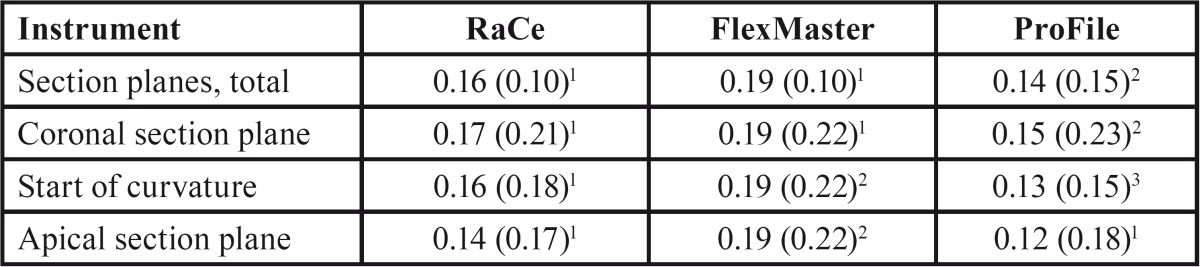
Postoperative differences of canal areas (mean ± SD/mm²).

Loss of working length was most pronounced in the FlexMaster group (0.13 mm ± 0.39 mm), followed by the RaCe (0.05 mm ± 0.15 mm) and ProFile (0.00 mm ± 0.00 mm) groups. These differences were, however, not statistically significant.

Overall, six RaCe files, three ProFile files, and one FlexMaster file fractured during the preparation of root canals. These differences were not statistically significant. The fractures occurred between the first and forth applications of the files and involved instruments of 2% conicity.

With regard to the preparation time, the lowest value was recorded for the FlexMaster group (795 s), followed by the RaCe group (825 s) and ProFile group (843 s). These differences were not statistically significant.

## Discussion

The present study in extracted human molars analyzed the shaping ability of three instrument types in the preparation of severely curved root canals with respect to postoperative cross-section of root canals, loss of working length, frequency of instrument fracture, and preparation time. In previous years, similar studies (16,17) were frequently analyzed by means of microscopic computerized tomography that provided a three-dimensional view of the complete tooth before and after preparation (18). Drawbacks of this method are its high costs and demand for large datasets (19). To ensure an equally detailed analysis of the root canals before and after their preparation with the two-dimensional Bramante technique, we used five (rather than the usual three) specimen sections in this study. Enlargement of the canal cross-sectional areas determined after root canal preparation provided indirect information on the shaping ability of the three instrument systems tested. Markedly enlarged cross-sectional areas indicated unwanted straightening of the original canal curvature.

The differences in cross-sectional area of root canals observed in this study indicated that use of the FlexMaster system led to the largest material loss, followed by RaCe and ProFile systems. This finding was confirmed for all canal sections tested, i.e., coronal section, section at the start of the curvature, and apical section. Because the postoperative cross-sectional areas differed only slightly from the preoperative areas and material loss was modest for all three systems, we conclude that all three instruments preserved the original root canal anatomy. This finding is in agreement with a published study comparing the shaping ability of RaCe, FlexMaster, and ProFile in simulated root canals (11). Moreover, our data are in concordance with those published by other authors who evaluated the various instrument types separately (7-9,16,20-22).

A critical aspect of our study design is the question whether the sole measurement of surface area differences before and after the preparation allows reliable conclusions regarding the preservation of the original canal anatomy. Because we did not determine the center of the original root canal surface, we cannot conclude definitively whether the area after the preparation was enlarged circumferentially or whether loss of material occurred mainly laterally. In addition, it may be questionable to what extent the randomized distribution of natural, non-standardized root canals with variable curvature, width, and length may affect the results in the three study groups.

For this reason, simulated synthetic root canals are frequently used in similar studies (11,23-26), which ensures high reproducibility of the experiment (3). However, because micro-hardness and abrasion properties of dentin and synthetic materials clearly differ, splinters of synthetics arising during their processing are of different size compared to those of dentin. These tend to block the apical region and cause difficulties in removing the synthetic chipping from the simulated canals (27). Thus, data obtained with simulated root canals translate less well into the clinical situation (3). For this reason, we used extracted human molars to ensure experimental conditions as close as possible to the clinical situation with respect to dentin hardness and the three-dimensional irregularities in the root canal.

During the study, six RaCe files, three ProFile files, and one FlexMaster file fractured. While the published fracture rates for FlexMaster (28,29) and ProFile instruments (30) compare well with our findings, RaCe files were reported to have a lower fracture rate in similar studies (16,21). Using the manual mode of the TriAuto ZX handpiece with switched-off torque-limiting auto-torque reverse function may be a possible reason, in addition to factors such as type and design of the instrument, method of use, angle of curvature, or different properties of dentin in extracted teeth. To our knowledge, no studies investigating the performance of RaCe instruments combined with the TriAuto ZX motor are available for comparison. Our findings confirm that a constant working length can be ensured with all three Ni-Ti instrument systems since we observed no or only negligible loss of working length in this study. In contrast, Schirrmeister *et al.* reported significantly better control of working length when using RaCe instruments compared with FlexMaster or ProFile instruments in a study involving simulated root canals (11).

In terms of the time required for the preparation of the root canals, our study did not find any significant difference between the various instruments. Because we used the same method and number of files of identical ISO size in all cases, the conditions for comparing the preparation times associated with the various instruments were ideal. In the study published by Schirrmeister *et al.* (11), the preparation times associated with the individual instruments differed markedly, with the procedure using RaCe instruments being significantly faster than that using either ProFile or FlexMaster instruments. However, the RaCe procedure involved only six files, while the procedure with ProFile and FlexMaster instruments involved nine and eight files, respectively.

## Conclusions

Based on the findings of the present *in vitro* study, we conclude that root canal preparation with the three instruments did not lead to any significant alteration of the original root anatomy or working length.
